# How Promising Are “Ultraprocessed” Front-of-Package Labels? A Formative Study with US Adults

**DOI:** 10.3390/nu16071072

**Published:** 2024-04-06

**Authors:** Aline D’Angelo Campos, Shu Wen Ng, Katherine McNeel, Marissa G. Hall

**Affiliations:** 1 Department of Health Behavior, Gillings School of Global Public Health, University of North Carolina at Chapel Hill, Chapel Hill, NC 27599, USA; adangelo-campos@unc.edu; 2 Carolina Population Center, University of North Carolina at Chapel Hill, Chapel Hill, NC 27516, USA; shuwen@unc.edu; 3 Department of Nutrition, Gillings School of Global Public Health, University of North Carolina at Chapel Hill, Chapel Hill, NC 27599, USA; 4 National Institute of Diabetes and Digestive and Kidney Diseases, Bethesda, MD 20892, USA; 5 Lineberger Comprehensive Cancer Center, University of North Carolina at Chapel Hill, Chapel Hill, NC 27599, USA

**Keywords:** ultraprocessed foods, warning labels, front-of-package labeling, food labeling, food policy, nutrition policy

## Abstract

High levels of food processing can have detrimental health effects independent of nutrient content. Experts and advocates have proposed adding information about food processing status to front-of-package labeling schemes, which currently exclusively focus on nutrient content. How consumers would perceive “ultraprocessed” labels has not yet been examined. To address this gap, we conducted a within-subjects online experiment with a convenience sample of 600 US adults. Participants viewed a product under three labeling conditions (control, “ultraprocessed” label, and “ultraprocessed” plus “high in sugar” label) in random order for a single product. The “ultraprocessed” label led participants to report thinking more about the risks of eating the product and discouraging them from wanting to buy the product more than the control, despite not grabbing more attention than the control. The “ultraprocessed” plus “high in sugar” labels grabbed more attention, led participants to think more about the risks of eating the product, and discouraged them from wanting to buy the product more than the “ultraprocessed” label alone. “Ultraprocessed” labels may constitute promising messages that could work in tandem with nutrient labels, and further research should examine how they would influence consumers’ actual intentions and behaviors.

## 1. Introduction

Ultraprocessed foods (UPFs), as defined by the Nova classification system, consist of formulations that contain little to no whole food ingredients, are assembled using intense industrial processing methods (including physical, chemical, and biological processing), and usually contain flavorings and cosmetic additives such as colorings, aromas, and emulsifiers [1]. The typification of UPFs is relatively recent, but these foods correspond to a large share of the energy intake globally. In the United States (US) and the United Kingdom (UK), over half of the daily calories consumed are estimated to come from UPFs, and in other high- and upper–middle-income countries, this figure ranges between 23% and 40% [2]. A substantive body of epidemiological evidence has shown associations between consumption of UPFs and all-cause mortality, cardiocerebrovascular diseases, hypertension, metabolic syndrome, type 2 diabetes, dyslipidemia, some cancers, and gastrointestinal disorders, among other conditions [3,4,5,6,7,8,9,10].

The mechanisms through which UPFs may lead to such health outcomes remain under investigation and debate. Although processing level and nutrient content are different concepts, UPFs tend to be high in specific nutrients of public health concern (i.e., added sugar, saturated and trans fats, sodium) and energy density, which partially explains their association with negative health outcomes [11,12]. However, several studies also demonstrate that these associations are not entirely explained by nutrient content [13,14,15,16,17,18]. The additives commonly present in UPFs, which are classified as xenobiotics, can cause alterations in the microbiota that lead to inflammatory responses [19,20,21,22,23,24,25]. Additionally, high levels of processing can alter the structural matrix of foods [2,26,27] and produce toxic compounds [28,29,30]—which, evidence indicates, can result in UPFs being poorly satiating and hyperglycemic [31,32,33,34,35,36]. These nutrient-independent mechanisms provide a rationale for public health efforts targeting UPF consumption in addition to efforts based on nutrient content.

Interpretive front-of-package labels (FOPLs) provide information about the nutritional quality of a food or beverage product through visual cues (e.g., symbols and graphics) as to how such information should be interpreted. A large body of evidence shows that FOPLs lead to an improvement in the nutrient content of consumers’ food and beverage choices [37,38,39,40,41,42,43]. As of 2023, 16 countries have implemented or approved legislation to implement mandatory FOPLs in an effort to improve population-level dietary quality [44], and the World Health Organization and World Cancer Federation also recommend FOPLs for their wide reach and low cost [45,46].

To date, all existing FOPL schemes are exclusively based on and convey information about products’ nutrient content, with no information on or consideration of foods’ processing level. However, the growing scientific recognition of the health implications associated with the processing level of foods has been spurring global momentum in discussions about UPFs—as evidenced, for instance, by the fact that “ultraprocessed” was one of Collins Dictionary’s top 10 words of 2023 [47]. In this context, experts and advocacy organizations have proposed incorporating labels identifying UPFs into FOPL schemes [48,49]. How such labels might be perceived by consumers is yet to be determined. This formative study aimed to examine whether front-of-package “ultraprocessed” labels are promising by examining message and effectiveness perceptions among a sample of US adults.

## 2. Materials and Methods

### 2.1. Participants

In July 2022, we recruited an online convenience sample of 600 adults through the Qualtrics Online Panel platform. Online convenience samples have been shown to often produce experimental effects that are similar in direction to those obtained from nationally representative samples [50,51]. This study was part of a larger survey about health behaviors with a predetermined number of participants. Participants were eligible if they were over 18 years old and resided in the US. Qualtrics tracks the location of IP addresses to ensure eligibility, uses a bot detection filter, and automatically deletes duplicate responses. Participants provided informed consent and received incentives in a reward type and amount set by the vendor. The study was approved by the University of North Carolina at Chapel Hill’s Institutional Review Board (#20-2338). The study design, measures, hypotheses, and analytic plan were registered before data collection at https://aspredicted.org/9tw9i.pdf (accessed on 5 January 2023).

### 2.2. Procedures

**Figure 1:**
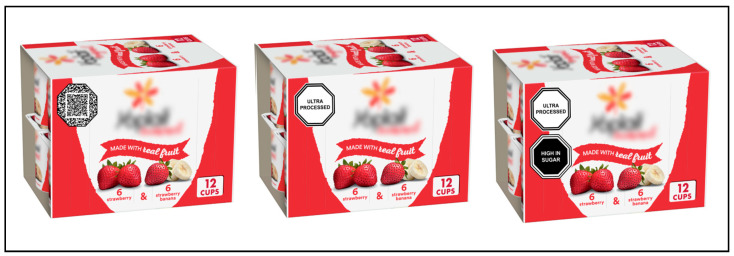
Study stimuli (control label, “ultraprocessed” label, combined “ultraprocessed” and nutrient labels, respectively).

This study used a within-subjects experimental design, which maximized power within our pre-determined sample size while still examining the perceived effects of messages [52,53]. Participants saw an image of a product under three different labeling conditions in random order: carrying a control label, carrying an “ultraprocessed” label, and carrying both an “ultraprocessed” label and a “high in sugar” label (Figure 1). We opted to examine an “ultraprocessed” label alone, given that this is a novel label whose independent influence on consumer perceptions has not yet been examined, as well as an “ultraprocessed” label combined with a nutrient label, since this is likely what consumers would encounter if “ultraprocessed” labels were incorporated into existing FOPL schemes.

We showed participants the same product in all conditions to control for brand preferences. Because previous evidence suggests that warning labels may be more effective at influencing perceptions of products originally perceived as healthy [54], we used a pack of yogurts—a product commonly perceived as beneficial to health [55], despite many varieties being ultraprocessed and high in sugar—from a popular US brand. We also used a QR code as the control label to account for any effects caused by the presence of a new label on the package and for any obscured branding. If scanned, this code led participants to a website that explained what a QR code is. Labels were octagon-shaped, since this design has been shown to perform the most consistently at improving consumers’ understanding of nutrient content and eliciting behavior change across a range of contexts [56,57,58,59,60]. The nutrient label used a black background with white text, mimicking the design used in countries like Chile and Mexico [44], while the “ultraprocessed” label used a white background with black text to distinguish processing level as a separate product dimension [49].

### 2.3. Measures

After seeing the stimuli under each labeling condition, participants answered three questions about the labels. First, the survey assessed how attention-grabbing participants perceived the labels to be and how much the labels led them to think about the risks of eating the product—two perceptions known to mediate the effects of messages on behavioral change [61] and commonly used in formative message-testing studies [62]. Finally, the survey assessed perceived message effectiveness (PME) with the item: “How much does this label discourage you from wanting to buy this product?” PME has been shown to predict actual message effectiveness (i.e., changes in behavioral intentions and behaviors), while remaining generally sensitive to detecting small differences between labels [63]. The single-item PME measure used in this study performs similarly to the original three-item scale [63]. Response options to all three questions were on a Likert-style scale, ranging from “Not at all” (coded as 1) to “Very much” (coded as 5). The survey also collected information on demographic characteristics.

### 2.4. Analysis

We conducted a post hoc power calculation to determine the largest effect size that we would be able to detect with our pre-determined sample size. Analyses indicated that, with our sample size of 600, 80% power, a critical alpha of 0.05, three repeated measurements, and an average correlation among repeated measures of 0.5, we would be able to detect a minimum effect size of d = 0.10 for each outcome. We descriptively compared results on our outcomes under each condition and calculated Cohen’s d as a standardized metric of effect sizes.

Next, we conducted mixed-effects linear regression models with random intercepts to statistically assess our effects while accounting for repeated measures within each individual. We used the “ultraprocessed” label condition as the reference in these models. Age, gender, race, ethnicity, education, and self-rated health status were also included as control variables in each of the models. We used complete case analysis to address any missing data, resulting in five observations being dropped for all outcomes in the main analyses. Analyses were conducted in G*Power version 3.1 and Stata/BE version 18 with a critical alpha of 0.05.

## 3. Results

Participants’ mean age was 44.6 years, and 74% identified as women. Around 76% of participants identified as non-Hispanic white, 15% as non-Hispanic Black, and 12% as Hispanic. About 29% obtained a high school diploma or less, and 8% had a graduate or professional degree (Table 1).

Since the key assumption underlying this study’s design is that the randomization of the order in which participants saw the conditions evenly distributes any carry-over effects due to label order among the sample [55], we conducted a χ^2^ test (not pre-registered) to ascertain whether the order of visualization of labeling conditions was evenly distributed. This test yielded null results (χ^2^ = 2.73, *p* > 0.05), indicating that the order in which participants viewed the different labeling conditions was evenly distributed across the sample.

**Figure 2:**
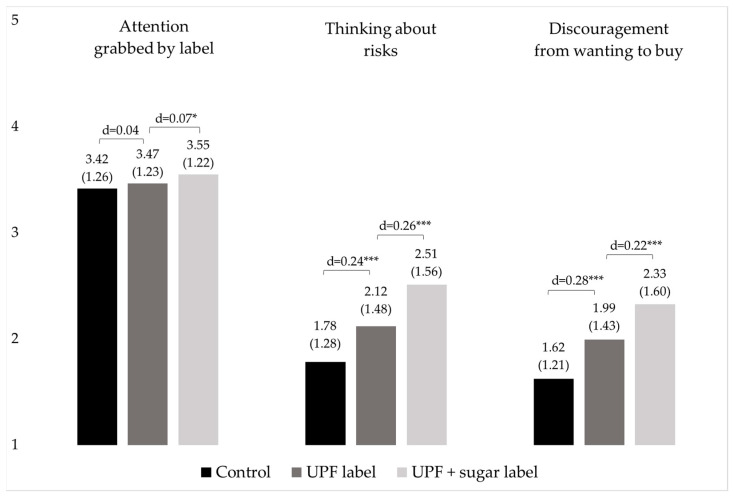
Mean (SD) attention grabbed by label, thinking about risks, and discouragement from wanting to buy, by experimental condition (*n* = 600); UPF = ultraprocessed food; 1 =”Not at all” 5 =”Very much”; * statistically significant at the 95% confidence level; *** statistically significant at the 99% confidence level.

Participants did not perceive the “ultraprocessed” label alone to be more attention-grabbing than the control label (d = 0.04, β = 0.04, *p* > 0.05). However, the combined “ultraprocessed” and “high in sugar” labels were perceived as more attention-grabbing (mean = 3.55) compared to the “ultraprocessed” label alone (mean = 3.47; d = 0.07, β = 0.08, *p* < 0.05; Figure 2, Table S1).

In terms of thinking about risks of eating the product, the “ultraprocessed” label performed better (mean = 2.12) than the control label (mean = 1.78, d = 0.24, β = 0.34, *p* < 0.01). In turn, when seeing the “ultraprocessed” and the “high in sugar” labels together, participants reported higher levels of thinking about risks (mean = 2.51) compared to the “ultraprocessed” label alone (d = 0.26, β = 0.4, *p* < 0.01; Figure 2, Table S1).

Finally, the “ultraprocessed” label elicited greater discouragement from wanting to buy the product (mean = 1.99) than the control label (mean = 1.62, d = 0.28, β = 0.38, *p* < 0.01). In turn, when seeing the “ultraprocessed” and the “high in sugar” labels together, participants reported higher levels of discouragement (mean = 2.33) compared to the “ultraprocessed” label alone (d = 0.22, β = 0.33, *p* < 0.01; Figure 2, Table S1).

## 4. Discussion

In this formative within-subjects online experiment with US adults, we examined how participants perceived a label informing them that a food is ultraprocessed, both on its own and in combination with a “high in sugar” label. Although the “ultraprocessed” label was not perceived as more attention-grabbing than the control, participants reported that it led them to think more about the risks of eating the product and discouraged them from wanting to buy the product more than the control. In turn, when combined with a “high in sugar” label, the “ultraprocessed” label had larger perceived effects than on its own.

The concept of UPF is complex, and the effect of an “ultraprocessed” label may depend on consumers’ understanding of it. A few qualitative studies in Latin America have found that the idea conveyed by the term “ultraprocessed” is reasonably well understood and associated with unhealthfulness [64,65,66], while studies in Europe have uncovered more variation in clarity about the concept [67,68]. Although, to our knowledge, no studies have assessed understanding of the term among US consumers yet, one study has shown that the perception of more processed foods as less healthful is lower among US consumers compared to consumers in Canada, Australia, the UK, and Mexico [69]. Our study found that an “ultraprocessed” label made US adults think more about the risks of eating the product and discouraged them from wanting to buy it compared to a control label. These initial findings suggest that “ultraprocessed” labels could constitute promising messages, and additional studies examining the labels’ actual effectiveness are warranted.

It is important to acknowledge that factors other than processing level play a role in consumers’ perceptions of different UPFs, with nutrient perceptions playing an especially important role [70]. Our findings corroborate this pattern: when both processing status and nutrient content were communicated through labels, perceived effects were larger than when only communicating processing status. These results suggest that “ultraprocessed” labels and nutrient labels could work together. Future research should further explore their joint effects, particularly investigating whether the addition of “ultraprocessed” labels to existing nutrient labels could amplify the overall effectiveness of labeling schemes. Lastly, it is worth noting that, should future research establish the effectiveness of “ultraprocessed” labels, robust criteria to assign individual products as UPFs would need to be developed before “ultraprocessed” labels became a viable policy option. Since the Nova framework does not currently provide easily operationalizable UPF assignment criteria, and products usually lack any information about processing methods, UPF identification has proven challenging even for experts [71].

This study’s strengths include the use of professionally developed stimuli featuring a real product and experimental design. However, this formative study also has limitations. As a small online experiment with a single product, we cannot establish how generalizable our findings would be in different settings and with different products. Future research should examine similar research questions using a broader range of products. Our study design only allowed us to assess the effects of our labeling conditions on message-related outcomes and not on actual effectiveness outcomes, which should be explored in future research. Our convenience sample also included a considerably larger proportion of women than men, which does not reflect the composition of the US population. However, it is worth noting that women are the usual grocery shoppers in a large majority of US households, so our findings may provide valuable insight into an intervention that could influence grocery shoppers [72]. Lastly, we did not measure or adjust results for some demographic characteristics such as rurality or anthropometric parameters, which would be worth including in future studies.

## 5. Conclusions

Existing evidence indicates that FOPLs can be an effective strategy to reduce consumption of unhealthful products. Our study suggests that “ultraprocessed” labels could work in tandem with nutrient labels by encouraging people to think about the health risks and possibly discouraging consumption of UPFs. Future research should explore if “ultraprocessed” labels affect consumers’ behavioral intentions and actual behaviors and whether they would offer additional benefits compared to nutrient labels already in place in many contexts.

## Figures and Tables

**Table 1:** Participant characteristics (*n* = 600).

		*n* (or Mean)	% (or SD)
Age		44.6	16.4
Gender			
	Man	154	26%
	Woman	443	74%
	Other	3	1%
Education			
	Less than high school	25	4%
	High school graduate (or GED)	152	25%
	Some college or technical school	169	28%
	Associate’s degree	89	15%
	Bachelor’s degree	119	20%
	Graduate or professional degree	46	8%
Race			
	White	455	76%
	Black or African American	87	15%
	American Indian or Alaska Native	10	2%
	Asian	19	3%
	Native Hawaiian or Other Pacific Islander	1	0%
	Other/Mixed race	28	5%
Hispanic ethnicity		70	12%
Household size (mean)		2.7	1.7
Household income			
	Less than $10,000	62	10%
	$10,000 to $14,999	38	6%
	$15,000 to $24,999	73	12%
	$25,000 to $34,999	85	14%
	$35,000 to $49,999	105	18%
	$50,000 to $74,999	119	20%
	$75,000 to $99,999	54	9%
	$100,000 to $149,999	42	7%
	$150,000 to $199,999	15	3%
	$200,000 or more	7	1%
Overall health			
	Poor	31	5%
	Fair	108	18%
	Good	240	40%
	Very good	145	24%
	Excellent	76	13%

## Data Availability

The de-identified dataset and code used in this study are publicly available at https://osf.io/XYDMQ/ (accessed on 5 January 2023).

## Supplementary Materials

The following supporting information can be downloaded at: https://www.mdpi.com/article/10.3390/nu16071072/s1, Table S1: Mixed-effect models assessing the effect of “ultraprocessed” labels on perceived attention, thinking about risks, and discouragement from buying (*n* = 595).
