# Supplementary material for: How Promising Are “Ultraprocessed” Front-of-Package Labels? A Formative Study with US Adults
Source: Nutrients. 2024 Apr 6;16(7):1072. doi: 10.3390/nu16071072 (PMC11013171; doi:10.3390/nu16071072)
Supplement: Supplementary file 1 [file nutrients-16-01072-s001.zip › nutrients-2926752-supplementary.pdf]

Table S1. Mixed-effect models assessing the effect of "ultraprocessed" labels on perceived attention, thinking about risks, and discouragement from buying (n=595)

	Attention			Thinking about risks			Discouragement from buying		
	**β**	**SE**	**95% CI**	**β**	**SE**	**95% CI**	**β**	**SE**	**95% CI**
**Label**									
UPF label (vs. control)	0.04	0.04	-0.03,0.11	0.34***	0.06	0.23,0.45	0.38***	0.06	0.27,0.49
UPF+Sugar (vs. UPF label)	0.08*	0.04	0.01,0.16	0.40***	0.06	0.28,0.51	0.33***	0.06	0.22,0.44
**Gender** (reference: male)	<0.01	0.11	-0.20,0.21	-0.04	0.11	-0.26,0.18	-0.12	0.11	-0.33,0.10
**Age**	<0.01	<0.01	-0.01,0.01	-0.01***	<0.01	-0.01,>-0.01	>-0.01*	<0.01	-0.01,>-0.01
**Higher education**	-0.17	0.10	-0.38,0.03	0.30***	0.11	0.09,0.51	0.31***	0.10	0.12,0.50
**Race** (reference: white)									
Black	0.24	0.13	-0.02,0.50	0.28*	0.14	0.01,0.55	0.23	0.14	-0.04,0.50
Other	0.03	0.17	-0.29,0.36	-0.01	0.17	-0.35,0.33	0.01	0.17	-0.33,0.34
**Hispanic ethnicity**	0.27	0.15	-0.02,0.56	0.38*	0.15	0.08,0.68	0.26	0.15	-0.04,0.56
**Good overall health**	0.16	0.11	-0.06,0.37	0.32***	0.11	0.10,0.55	0.29*	0.11	0.06,0.51

Note: UPF=ultraprocessed food
*Statistically significant at the 95% confidence level
***Statistically significant at the 99% confidence level
